# Novel Technologies for Seaweed Polysaccharides Extraction and Their Use in Food with Therapeutically Applications—A Review

**DOI:** 10.3390/foods11172654

**Published:** 2022-09-01

**Authors:** Silvia Lomartire, Ana M. M. Gonçalves

**Affiliations:** 1 University of Coimbra, MARE–Marine and Environmental Sciences Centre/ARNET–Aquatic Research Network, Department of Life Sciences, Calçada Martim de Freitas, 3000-456 Coimbra, Portugal; 2 Department of Biology and CESAM, University of Aveiro, 3810-193 Aveiro, Portugal

**Keywords:** seaweed compounds, nutraceutical, functional food, hydrocolloids extraction

## Abstract

The use of seaweed for therapeutic purposes is ancient, but only in the last decade, with advanced technologies, has it been possible to extract seaweed’s bioactive compounds and test their potential properties. Algal metabolites possess nutritional properties, but they also exhibit antioxidant, antimicrobial, and antiviral activities, which allow them to be involved in several pharmaceutical applications. Seaweeds have been incorporated since ancient times into diets as a whole food. With the isolation of particular seaweed compounds, it would be possible to develop new types of food with therapeutically properties. Polysaccharides make up the majority of seaweed biomass, which has triggered an increase in interest in using seaweed for commercial purposes, particularly in the production of agar, carrageenan, and alginate. The bio-properties of polysaccharides are strictly dependent to their chemical characteristics and structure, which varies depending on the species, their life cycles, and other biotic and abiotic factors. Through this review, techniques for seaweed polysaccharides extraction are reported, with studies addressing the advantages for human health from the incorporation of algal compounds as dietary supplements and food additives.

## 1. Introduction

The use of seaweed (also called macroalgae) for medicinal purposes sees its roots in Asian countries, which, since ancient times, saw and explored the incredible benefits of seaweeds and algae, and introduced them into traditional medicine practices. Seaweeds are classified in phylum Ochrophyta (Phaeophyceae, brown algae), phylum Rhodophyta (red algae), and phylum Chlorophyta (green algae). Each group presents diverse bioactive compounds, with several properties and mechanism of actions. Seaweed metabolites, besides providing protective actions, possess a high nutritional content, and they assure several advantages for people’s health. Over the years, many researches, through pre-clinical and clinical studies, have confirmed the positive effects of seaweed extracts, exhibiting antibacterial [1], antiviral [2] anti-inflammatory, anticoagulant, antithrombotic [3], anticoagulant [4], and antitumoral effects [5].

The new challenge is to develop strategies to combine the benefits of seaweed with food products, to have in the market not only sustainable and natural products, but also products with therapeutical properties. Seaweed hydrocolloids such as agar, carrageenan (abundant in Rhodophyta), and alginates (abundant in Phaeophyceae), are abundantly extracted and employed in food (gelling agents), pharmaceutical products (dressings, coatings of medicaments, stabilizers), biotechnology (culture medium, the Petri dishes), and cosmetics (body lotions, soaps, shampoos, toothpaste) [6,7]. Therefore, by investigating their beneficial properties it might be possible to develop specific functional foods adaptable for diverse demands [8].

Every day, the global commercial seaweed market acquires value; in recent years, the inclusion of hydrocolloids in the food, pharmaceutical, and other industries, has increased. Since seaweed can grow in any sort of aquatic environment, and are simple to grow, many industries throughout the world are already investing in their production. These factors include sunlight, natural or artificial aeration, and nutrient-rich seawater. The consummation of natural products for health or ethical reason is increasing, therefore, seaweed in human food, animal feed, and pharmaceutical products, has largely expanded over the years (https://www.fortunebusinessinsights.com/industry-reports/commercial-seaweed-market-100077, accessed on 10 June 2022).

Nevertheless, seaweed species, environmental factors, extraction techniques, and treatment procedures, have all had an impact on the physicochemical quality of compounds [9,10]. As a result, a thorough understanding of these variables will help us identify the most effective methods for obtaining high-quality phycocolloids, for specific purposes.

Analyzed throughout the present review, hydrocolloid extraction techniques are discussed, with a specific mention of novel extraction methodologies, which are eco-sustainable, and need to be further evaluated and ameliorated in order to substitute new extraction techniques for traditional ones that, at present, have several drawbacks. 

## 2. Therapeutic Properties of Seaweed Compounds

Seaweeds are ubiquitous, and to survive in harsh conditions they need to develop defense mechanisms; therefore, seaweeds have developed several metabolites that help their survival. Furthermore, seaweed bioactive compounds involved in the mechanisms of defense possess interesting properties that may ameliorate human health conditions. 

Antioxidant activity, for example, is fundamental to prevent human cells and organs from the effects of oxidative stress, due to the presence of reactive oxygen species (ROS) and free radicals. Oxidative stresses caused by ROS may lead to harmful pathologies, such as cancer, diabetes, neurodegenerative and cardiovascular diseases. In seaweed, antioxidant activity is expressed by pigments as chlorophylls, xanthophylls (fucoxanthin), carotenoids, vitamins (vitamins B1, B3, C, and E), vitamin precursors, and phenolic compounds such as polyphenols, and flavonoids [11,12,13]. Moreover, the incorporation of antioxidant compounds in food will provide its spoilage. 

Seaweeds also possess antimicrobial activities. According to Rajauria et al. [14], the antibacterial activity is triggered by algal polyphenols such as tannins, quinones, flavones, flavonols, phlorotannins, and flavonoids. *Himanthalia elongata* methanolic extracts have shown antibacterial efficacy against pathogenic bacteria (*Listeria monocytogenes* and *Salmonella abony*), and food deterioration bacteria (*Enterococcus faecalis* and *Pseudomonas aeruginosa*) [14]. Gram-positive and gram-negative bacteria have both been demonstrated to be resistant in the antimicrobial effects of terpenes, phlorotannins isolated from *Ecklonia kurome*, *Ecklonia cava*, and *Fucus vesiculosus* [15]. Algal polysaccharides exhibit antimicrobial activity, in addition to polyphenols, by identifying and attaching to the glycoprotein receptors on bacterial surfaces, with the consequential disruption of the bacterial cell. Moreover, to survive several viral attacks in hostile environments, seaweeds have developed antiviral compounds that stop a virus from entering the host cell or stopping its reproduction cycle at a specific stage. Alginates, carrageenan, agarans, DL-hybrid galactans, laminarans, fucans, and fucoidans, are sulphated polysaccharides found in seaweeds that have been discovered to prevent the replication of a diversity of enveloped viruses [16].

Biological activities performed from carrageenan depend on molecule size, sulphation degree, and glycosides branching. Because of their physical qualities, such as thickness, gelling, and stabilizing capabilities, most carrageenans used in the manufacturing of functional food have a high molecular weight fraction (HMWF). However, because of their high viscosity, these qualities may cause problems in non-food applications. As a result, converting carrageenan’s HMWF to a low molecular weight fraction (LMWF) could improve bioavailability, and enhance carrageenan’s potential applications in the nutraceutical, pharmacological, and biological industries [17].

It has been already demonstrated that LMWF carrageenan showed interesting biological activities compared to HMWF, such as being antioxidant, antiproliferative, and antiviral. Common techniques used to depolymerize carrageenan are microwave, sonication, irradiation, and oxidation utilizing H_2_O_2_ [17]. In general, low molecular weight sulphated polysaccharides provides interesting biological activities. For instance, Chen et al. [18] revealed the action of seaweed LMWF sulphated polysaccharides that generates the immunostimulant activity against S180 tumors in mice. Another case study demonstrated that LMWF fucans from *Ascophyllum nodosum* showed a higher inhibition of cell growth action on fibroblast cell lines CCL39 [19]. Similarly, low molecular weight fucoidans from *Fucus vesiculosus* inhibited the proliferation of B16 melanoma cells, Lewis lung cancer, and Sarcoma 180 cell lines, showing greater anti-angiogenesis activity [20]. Sulphate content was high in both fucans and fucoidans, with increased antiproliferative activity, showing that sulphate content influenced anticancer action.

The therapeutic activities of seaweed bioactive compounds are multiple and depends on the characteristics of seaweeds and their compounds, but also depend on the extraction processes. The nutraceutical industry provides the development of therapeutical food made by the incorporation of seaweed compounds, which provide advantages to our health. Among seaweed polysaccharides, carrageenan, agar and alginate are the most involved in nutraceutical and the food industry. 

### 2.1. Commercial Seaweed Hydrocolloids

Sulphated polysaccharides are negatively charged polysaccharides found in seaweed cell walls, which are mostly made up of cellulose and hemicellulose, and have a high carbohydrate content but few calories and fats. Non-toxicity, antimicrobial, antioxidant, anticoagulant, anti-inflammatory, antitumoral, and antiviral properties, make sulphated polysaccharides potential candidates for medical applications and nutraceutical foods [21,22]. Hydrocolloids commonly extract agar, carrageenan, and alginates, from seaweed.

#### 2.1.1. Agar

D-galactose and 3,6-anhydro-L-galactose repeating units constitute agar (or agar-agar) molecules. There are not too many variants of this chemical, and its ester sulphate content depend on species by species. Agarose, a linear and neutral polysaccharide, and agaropectin, constitute the two groups of polysaccharides that forms agar structure [23,24,25].

**Figure 1:**
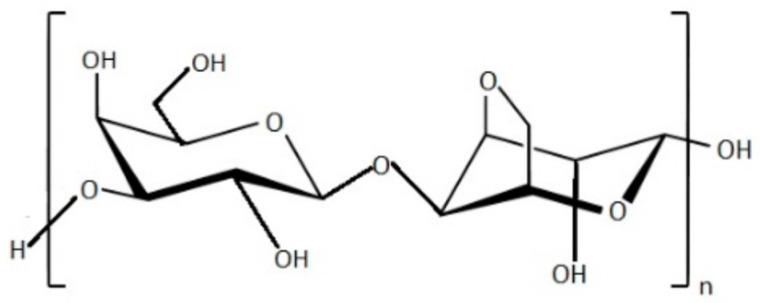
Chemical structure of agarose polymer.

Because of its gelling ability, agarose is frequently utilized in pharmaceutical, cosmetic, and medicine applications, and it also possesses good biofilm properties [26] (Figure 1). Due to its capacity to serve as stabilizers, emulsifiers, and thickening agents, agar is commonly used in the market food processing business. It is used in the food industry for a variety of purposes, such as to improve the texture of dairy products. It is also a clarifying agent for wines, especially plum wines, in the alcoholic industries [27]. Compared with carrageenan and alginate films, agar-based gels are typically transparent and tight; the addition of sugars might add strength to them [28], indeed agar is widely employed to produce desserts, jams, jellies, and bakery products. Compared to carrageenan and alginate films, agar films have a poor tensile strength and water vapor permeability, but they show superior elasticity and double the elongation value of κ-carrageenan film [29,30]. Agar has significant biological effects because it inhibits the growth of tumors, diminishes oxidative stress, and lowers levels in diabetic patients. Other uses include the production of capsules and cell culture medium [31]. 

#### 2.1.2. Carrageenan

**Figure 2:**
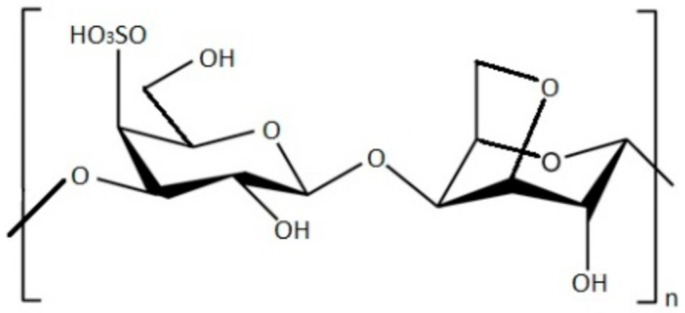
Chemical structure of κ-carrageenan.

Carrageenan is a linear polymer, primarily found in red algae, that is made by alternating sulfated or non-sulfated galactose units joined by α-1,3-glycosidic and β-1,4-galactose linkages (Figure 2). Depending on where the sulfate group attaches to the galactose unit, carrageenan can be classified into many forms; in nature, they are typically hybrids [32]. The following are the names of the pure carrageenans: κ-carrageenan, ι-carrageenan, λ-carrageenan, γ-carrageenan, ν-carrageenan, ξ-carrageenan, and µ-carrageenan [33]. 

Carrageenans are frequently used in the food business because of their gelling, thickening, and stabilizing abilities [34]. The commercial versions of ʎ-, κ- and ɩ-carrageenan have received approval as food supplements from the Food and Drug Administration (FDA) and the European Food Safety Agency (EFSA) [35]. Carrageenan’s biological potential has been studied in the medical field over the past few decades, and results have been promising. Indeed, researchers discovered that carrageenan has anticoagulant, antithrombotic [36], antivirus [37], antiproliferative [38], and antioxidant properties [39].

Carrageenans are water soluble, yet the factors that affect this solubility in water include temperature, pH, medium ionic strength, and the presence of cations. Their hydrophobicity is mostly derived from the 3,6-anhydro-D-galactopyranose units, while their hydrophilicity is determined by the sulfate and hydroxyl groups [34].

κ-carrageenan and ι-carrageenan are both stable at room temperature, while λ-carrageenan possesses normal nongelling property and is a unique carrageenan, naturally occurring as cold-water soluble carrageenan. Phycocolloids’ ability to produce gels and maintain strength is improved after the enrichment of gel with cations, as the findings of Robal et al. [40] confirmed for κ-carrageenan and ι-carrageenan.

#### 2.1.3. Alginate

*Ascophyllum nodosum*, *Macrocystis pyrifera*, *Durvillaea antarctica*, *Sargassum turbinaroides*, *Lessonia nigrescens*, and *Ecklonia maxima* [41] are the main sources of alginate.

**Figure 3:**
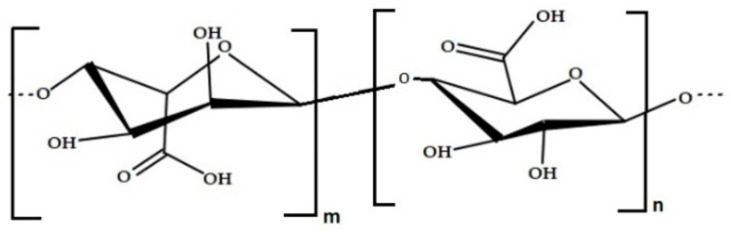
Chemical structure of alginic acid.

Alginates derive from derivates of alginic acid and salts [34,42]. Their structures are composed by anionic linear polysaccharides, with the ability to model films safe for consumption. Alginate is made up of alginic acid polymers that have 1,4 linkages between the monomer units of β-D-mannuronic acid (M) and α-L-guluronic acid (G) [43] (Figure 3). It is frequently used in foods and medications due to its strong stabilizing and thickening qualities.

A high guluronic acid content produces a gel with enhanced gelling and elastic characteristics, because the physicochemical and mechanical characteristics of gels made with alginic derivates vary depending on the M/G ratio and structure length. Low M/G ratios, on the other hand, produce strong, brittle gels with exceptional heat stability, but they also exhibit syneresis during freezing processing, that is, the extraction or expulsion of a liquid phase from a gel phase [34,42]. Alginates bind the alkaline metals calcium and sodium ions, increasing the rigidity and stability of the gel matrix [41,43]. Paula et al. [44] investigated the mechanical properties of glycerol-plasticized edible films made with different phycocolloids; when compared to κ-carrageenan, the latter showed greater tensile strength, elasticity, moisture permeability, and reduced opacity, while alginate films showed more transparency [44].

## 3. Extraction Processes for Seaweed Hydrocolloids

In traditional extraction methods, maceration occurs in water at high temperatures. The extraction procedures differ by sources and application of polysaccharides. Cleaning the seaweed of epiphytes, debris, salts, pollutants, sand, and toxins, is necessary prior to extraction. Alkali pre-treatment of agar and carrageenan improves the gelling characteristics by reducing unstable sulphate molecules into 3,6-anhydro-L-galactopyranose (3,6-AG) [42]. In order to increase alginate yield and remove color pigments from seaweed tissue, alginate is pre-treated with formaldehyde. It is also pre-treated with hydrochloric acid (HCl) in order to “clarify” the phenolic compounds and formaldehyde residue, and to encourage the conversion of insoluble salts (calcium, magnesium, etc.) into soluble salts [45,46,47,48,49].

Hot water extraction is used for carrageenan and agar, followed by alkali extraction, to obtain molecules with the desired characteristics and functions by manipulating conditions such as time, pH, solvent concentration, and temperature, etc.

The choice between alkaline extraction, or water extraction for agar extraction, depends on the species. For example, alkali treatment is necessary for *Gracilaria* spp. in order to produce 3,6-anhydrogalactose, which is accountable for forming a strong agar gel, but it is not necessary for *Gelidium* spp. [50]. The alkaline treatment, however, can be used to increase the gel strength of carrageenan even though it is not necessary for carrageenan extraction [51].

The temperature at which hydrocolloids are extracted varies depending on the hydrocolloids of interest; for instance, agar extraction is performed at temperatures higher than 85 °C. Carrageenans can dissolve in either cold or hot water due to their chemical composition; however, pH should be maintained above the pKa value of alginate (between 3.4 and 4.4) because pH is the most crucial solubilizing parameter for alginate extraction [34]. 

**Figure 4:**
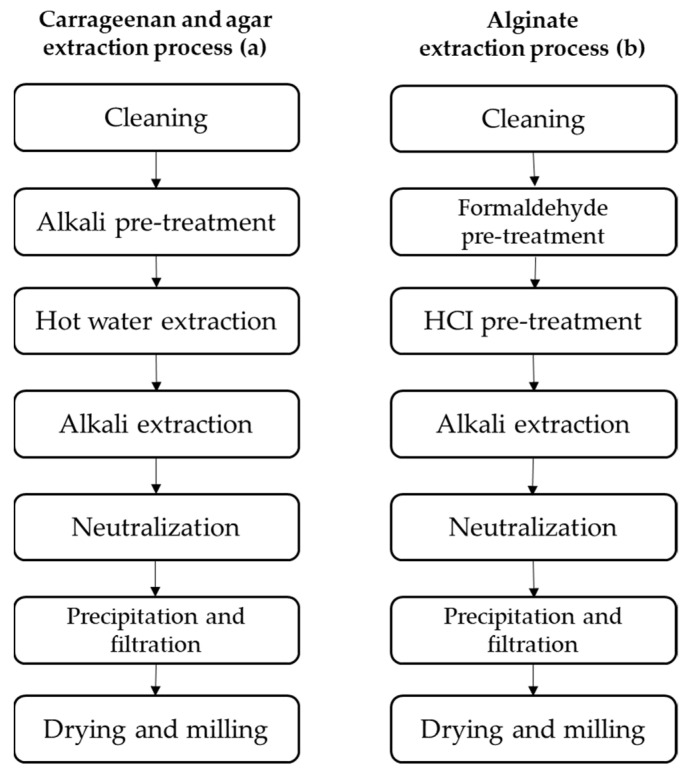
Extraction processes for commercial macroalgal hydrocolloids: (**a**) carrageenan and agar; and (**b**) alginate.

Alginate extraction is performed only with alkali extraction. Later, all phycocolloids are then neutralized by eliminating chemicals and solvents in excess; residuals are then removed using precipitation and filtering, leaving just the pure compound; finally, grinding and drying are carried out to produce the finished products, which are dried, cleansed, and prepared for use in commerce (Figure 4) [42].

Traditional approaches for processing seaweed to generate hydrocolloids necessitate an intensive use of time and solvents. Innovative technologies for hydrocolloid extraction and production are currently being investigated at various stages of macroalgae processing, with the goal of improving the yields of valuable compounds, while improving the productivity of actual industrial procedures and minimizing, or avoiding, the use of organic solvents [52].

### 3.1. New Extraction Methods

Common hydrocolloid extraction has several drawbacks, including huge time, energy, and water consumption. Furthermore, many chemical solvents are employed to achieve an optimal yield, some of which are health concerns; due to poor regulation throughout the entire manufacturing process and discharge, the use of chemicals may pose a major threat to both human health and the environment [53,54]. A disadvantage of the old extraction method is the high price of solvents used during carrageenan precipitation to create refined carrageenans [53].

Traditional extractions processing can result in enormous amounts of hazardous waste. Furthermore, if the solvent being used is noxious, additional processing steps are required to meet alimentary and pharmaceutical sector rules to use extracted compounds incorporated in safe products [55]. With this concern, sustainable extract technologies are currently a primary issue in natural product recovery research and development [42,55]. The use of eco-friendly solvents, such as ionic liquids, eutectic solvents, surfactants, or solvents from biological origin, is an alternative way to minimize the adverse effects of hazardous chemicals used in the extraction process. For example, deep eutectic solvents are available from natural chemicals. Smith et al. [56] divided deep eutectic natural solvents into four categories: (a) mixtures of organic salts and metal salts; (b) mixtures of organic salts and metal hydrates; (c) mixtures of organic salts and hydrogen bond donors; and (d) mixtures of metal chlorides and hydrogen bond donors. When extracting seaweed polysaccharides, deep eutectic natural solvents are a more environmentally friendly choice than organic solvents because of their lower price, biopolymer dissolving capabilities, biodegradability, non-toxicity, polarity, and recyclability [57]. Natural deep eutectic solvents based on choline chloride, lactic acid, betaine, and glucose, have been already used to extract phlorotannins from brown algae *Fucus vesiculosus* and *Ascophyllum nodosum*. Extraction yields of phlorotannins achieved was around 60–72%. Nie et al. [58] proposed the extraction of polysaccharides from *Sargassum horneri* using ultrasonic extraction and deep eutectic solvents composed of choline chloride, 1,2-propanediol, and water. The results indicated that deep eutectic solvents had stronger protein and CaCO_3_ removal ability than that of a conventional hot water extraction method, suggesting these solvents were good alternatives [58]. A solid-phase extraction (SPE) of fucoidan and laminarin was performed on kelps. The deep eutectic solvent prepared by choline chloride and urea had the best extraction efficiencies for fucoidan and laminarin (95.5% and 87.6%, respectively) [59].

Three different deep eutectic solvents prepared by the complexation of choline chloride with urea, ethylene glycol, and glycerol, as well as their hydrated counterparts, were used for the selective extraction of ĸ-carrageenan from *Kappaphycus alvarezii,* and the obtained yield was compared with ĸ-carrageenan extracted using a conventional method. It was inferred from the studies that the physicochemical as well as rheological properties of the polysaccharide, obtained using eutectic solvents, were superior in comparison to the ĸ-carrageenan obtained using water as solvent [60].

Alternative extraction and processing methods include microwave-assisted extraction (MAE), ultrasound-assisted extraction (UAE), high-pressure technique, and enzyme-assisted extraction (EAE). Some of these techniques have already been applied to the extraction of bioactive chemicals from plants [61,62]. Nevertheless, all of these processes have advantages and disadvantages in terms of time, expenses, and production output [42].

#### 3.1.1. Microwave-Assisted Extraction (MAE)

Microwave techniques are based on the use of electromagnetic radiation on a sample matrix at varying frequencies (0.3–300 GHz) and wavelengths (1 mm to 1 m) [63]. Cell walls are broken by the microwaves’ uniform heating of the matrix [64,65]; as a result of the increased pressure brought on by moisture evaporation, the matrix increased porosity, solvent penetration, and the release of solutes from cells [66]. Additionally, microwaves can be used in closed or open vessels (at atmospheric pressure), allowing for the application of high pressure, and reaching solvent boiling points at lower temperatures than those in open vessel format [63,67]. While using MAE, some of the crucial elements to take into account are: the frequency; power; extraction time and pressure; solid/liquid ratio; solvent concentration and properties; matrix characteristics; temperature; and the number of extraction cycles. It provides numerous pros, including consistency; efficiency; the capacity to selectively and locally heat raw materials; enhanced mass transferring and tissue degradation; reduced extraction time and energy; less solvent consumption; low cost; high extraction rate; and good product quality. Microwave techniques can be utilized in fresh biomass and are now applied to extract various compounds (polysaccharides, phenolics, etc.) from seaweed [42,55,68]. Magnusson et al. [69] found that microwave techniques increased polyphenol yields from *Carpophyllum flexuosum* by 70% when compared to traditional extraction, and Boulho et al. [70] found that MAE increased carrageenan yields from *Solieria chordalis* by 20%. However, it is challenging to extract some thermolabile chemicals because in these technologies rapid heat increases (i.e., fatty acids, pigments, proteins) [55]. The open and closed systems are the two types of systems used for MAE. The open system is less expensive, completely automated, and operates at air pressure, eliminating the risk of ignition. However, it has less precision, it is unable to operate with multiple samples at the same time, and it requires a longer extraction time [42,63]. The closed system is employed at high pressures and temperatures, posing an explosive danger. Fucoidan, from *Fucus vesiculosus,* have been extracted by microwave-assisted extraction (MAE), and degradation (%), total sugar yield (%), and SO_3_ content (%) were determined with diverse experimental conditions. MAE at 120 psi, 1 min, using 1 g alga/25 ml water, was the best condition for fucoidan recovery; therefore, this method has been approved, also due to the short extraction times and non-corrosive solvents, resulting in reduced costs and being an environmentally friendly technique [65]. This technique also allowed for the extraction of phlorotannins from *Fucus vesiculosus* [71].

This method has been also used to extract carrageenan from *Solieria chordalis* (Rhodophyta) to investigate carrageenan yield, physicochemical properties, and antiviral activity; but no significant differences in the carrageenan yield were observed between MAE and the conventional method, under alkaline conditions [70].

Sulfated polysaccharides (fucoidan) from brown seaweed *Ascophyllum nodosum* were extracted by MAE. Different conditions of temperature (90–150 °C) and extraction time (5–30 min) were evaluated and the optimal fucoidan yield was 16.08%, obtained from 120 °C for 15 min extraction, showing that MAE is an efficient technology to extract sulfated polysaccharides from seaweed [72].

#### 3.1.2. Ultrasound-Assisted Extraction (UAE)

The use of sound frequencies above the audible frequency range (>20 kHz) and below microwave frequencies (≤10 MHz) that propagate on samples as compression and rarefaction waves, is the foundation of ultrasound technology. These technologies can be classified into low-intensity (less than 1 W/cm^2^) and high-intensity sonication (between 10 and 1000 W/cm^2^), with the latter being the most popular method for obtaining nutritive compounds [73]. When an ultrasound wave passes through a solvent, it causes acoustic cavitation and the development of cavitation bubbles, increasing the surface of contact between the liquid and solid phases. This provides for a better solvent penetration and faster solute diffusion into the matrix. Solid-liquid suspensions produce asymmetrical bubbles, which capture vapor from the solvent, causing implosion and creating mechanical energy through microturbulence, which breaks the algal cell wall and optimizes extraction efficiency [42,55,63,68]. 

Pressure, frequency, wave intensity, temperature, solvent viscosity, and surface tension, are all variables that might affect the extraction process [55]. UAE increases extraction yield, while decreasing extraction time. In comparison to traditional approaches, this extraction is effective, eco-friendly, easy, and inexpensive. The food sector has adopted this novel extraction to extract a large variety of compounds due to the low cost of equipment maintenance. Moreover, the equipment is small in size, allowing to minimize the space used, and it can direct scale-up to industrial scale [42,55,68]. There are only a few investigations on the extraction of chemicals from marine seaweed employing UAE, but they have produced some extremely encouraging findings [42,55,68]. When compared to conventional procedures, carrageenan yield and purity are higher with UAE, according to Rafiquzzaman et al. [74], while Kadam et al. [75] found that laminarin extracted using UAE had a higher yield and antioxidant properties. The main disadvantage is that, due to the high cost of energy and equipment, UAE needs a large amount of capital to get started on an industrial scale, and its application is still limited. However, used alone, or combined with other methods, UAE can be an innovative technology [55,68].

Alginates from brown seaweed *Sargassum binderi* and *Turbinaria ornate,* and carrageenan from red seaweed *Kappaphycus alvarezii* and *Euchema denticulatum,* were extracted with the assistance of ultrasound. The extracted polysaccharides represented up to 55% of the seaweed’s dry weight and were obtained in a short time (15–30 min), compared to 27% in 2 h for conventional extraction. The authors saw that the extraction technique did not affect the chemical structure and molar mass distribution of alginates and carrageenan. Moreover, UAE reduced extraction time [76]. Extraction of seaweed compounds performed with UAE has been also investigated on yields of bioactive compounds (total phenolics, fucose, and uronic acid) from *Ascophyllum nodosum,* with great results regarding compounds yields [75], as well as for extraction of alginates from *Ascophyllum nodosum* [77]. The effects of UAE extraction variables, including temperature (40–60 °C); extraction time (50–80 min); ethanol concentration (0–60%); sample-to-solvent ratio (1–5 g/100 mL) on the total phenolic content (TPC); DPPH radical scavenging activity (DRSA); and ferric reducing antioxidant power (FRAP); were determined for *Padina australis*. The study revealed that the optimal UAE conditions were determined to be: ultrasonic temperature of 60 °C; ultrasonic time of 60 min; solvent concentration of 60% (*v*/*v*) aqueous ethanol and sample-to-solvent ratio of 1 g/100 mL; considering UAE a good method for saving time and solvents [78].

#### 3.1.3. High-Pressure Technology

These extraction techniques work by providing pressure to the solvent in the 3.5–20 MPa range, which keeps it liquid at temperatures above its regular boiling point. A greater pressure allows more solvent to permeate the sample matrix by lowering the viscosity and surface tension of the solvents and raising the permeability of the cell walls [62]. When water is the solvent, pressurized solvent extraction (PSE) employs temperatures and pressures between 50 and 300 °C, and 35 and 200 bar, respectively. The solvent reaches temperature and pressure that are beyond its regular boiling point but below the critical point, allowing it to stay liquid. Additionally, density, viscosity, and the surface tension of some solvents, decrease with increasing temperature, enabling quicker mass transfer and extraction yields [42,55,63]. Although water is often utilized as solvent, since it does not produce hazardous pollutants, different solvents might be employed [42,63]. The combination of high pressure and temperature permit increased desorption of target chemicals from the extraction matrix, as well as solvent solubility and diffusion, resulting in improved extraction kinetics. As a result, PSE has various advantages, including high extraction efficiency, less solvent consumption, quick extraction time, and non-risky extraction [42,55]. The extraction of labile compounds must be short, as they might degrade with increased pressure and temperature. Despite the considerable application of this extraction method, there is a scarcity of publications on seaweed extractions employing PSE. Phenolics, polysaccharides, and amino acids, are among the compounds that can be extracted, having powerful applications in several industrial sectors [42,55].

When phenolic compounds from the brown seaweed *Fucus vesiculosus* were extracted with pressurized liquid extraction (PLE), and at optimal conditions (137.18 °C, 58.65% *v*/*v* ethanol in water and 4.68 min extraction time), results yield 31.16% of dry basis. The total phenolic content value and radical scavenging activity of the PLE extract were insignificantly different from the one obtained from conventional solvent extraction, which proved new method advantages in terms of shorter time and less solvent requirement [79].

Pressurized liquid extraction (PLE) was used to obtain antiviral compounds from the edible seaweed *Himanthalia elongata*. The antiviral properties of PLE extracts (hexane, ethanol, and water) were evaluated against herpes simplex virus type 1 (HSV-1) at different stages during viral infection. The results demonstrated that PLE was an appropriate technique to obtain antiviral agents from *Himanthalia elongata* [80]. This technique has also been investigated to extract compounds from red seaweed *Kappaphycus alvarezii*, considering this method approved for the extraction of carrageenan [81].

#### 3.1.4. Enzyme-Assisted Extraction (EAE)

Enzymatic extraction involves hydrolysis of seaweed biomass by introducing enzymes that break the backbone of the algal cell walls polysaccharides. Cellulase, pectinase, glucosidase, xylanase, or gluconase, are commonly used enzymatic treatments in macroalgae, with the goal of increasing the extraction of components retained by the presence of hydrogen or hydrophobic interactions in the cells [64]. Temperature, pH, substrate-to-enzyme ratio, solvent, and agitation, are all important factors to consider when performing necessary reactions to improve the extraction efficiency [63]. 

EAE benefits include: low cost; transforming molecules that are insoluble in water into molecules that are soluble in water; better efficiency and specificity in target end products; process scalability; avoiding the use of any dangerous chemicals or organic solvents; and shorter extraction duration [55,63,82]. To date, there has been little research on the use of EAE in seaweed. Alginates, and other polysaccharides from *Cystoseira trinodis,* have also been obtained using enzyme-assisted pre-treatments [83]. When compared to conventional approaches, scientists found that an enzymatic pre-treatment increased the total antioxidant activity, while the yields of alginates and fucoidan were not significantly contrasting between the two procedures, according to the study [83]. *Sargassum muticum*, *Osmundea pinnatifida*, and *Codium tomentosum,* underwent EAE extraction and presented the highest levels of nitrogen and total phenolics, as well as for levels of sulphated polysaccharides, and the levels of prebiotic activity, respectively. The EAE extracts of *Codium tomentosum* and *Osmundea pinnatifida* had a 38–49% inhibitory potency against -glucosidase [84]. In order to extract carrageenan with abundant extraction yield and strong gelling characteristics, EAE was also used [60]. Other investigations revealed that EAE maintains the target compounds’ structural integrity (i.e., proteins, ulvans, lipids, fucoidans, etc.). The products were also highly bioactive and suitable for the cosmetic, nutraceutical, and pharmaceutical sectors [55,82,85].

EAE was used as a tool to extract bioactive compounds from seven brown seaweeds from the Kuwait coast, and characterization of the active extracts. Among the seven species of brown seaweeds studied, the enzymatic extracts obtained from *Sargassum boveanum*, *Sargassum angustifolium*, and *Feldmannia irregularis,* showed high antioxidant activity in different assays. Though antimicrobial activities of the enzymatic extracts were low, Flavourzyme resulted in a greater number of seaweed extracts with antimicrobial activity against foodborne pathogens. The results of the study showed that enzyme-assisted extraction could be useful to obtain seaweed extracts with specific bioactivity [86]. EAE of *Ulva armoricana* (Ulvales, Ulvophyceae) compounds reports antiviral and antioxidant activities of *Ulva armoricana* extracts, considering enzyme-assisted extraction a good method for this species [82].

Protein extracts from the brown seaweed *Macrocystis pyrifera* and the red seaweed *Chondracanthus chamissoi* were obtained by EAE using cellulase to enhance the protein extraction yields. The comparison of protein content obtained by enzymatic and non-enzymatic methods suggests that the disruption of the cellulase-sensitive carbohydrate matrix increases protein content of the extract. The protein extraction yields were 74.6% for *Macrocystis pyrifera* (18 h, 1/10 enzyme/seaweed ratio) and 36.1% for *Chondracanthus chamissoi* (12 h, 1/10 enzyme/seaweed ratio). Both protein extracts showed antioxidant activity and *Macrocystis pyrifera* protein extract showed a potential antihypertensive activity [87].

### 3.2. Traditional Extraction Techniques vs. Alternative Extraction Techniques

Traditional extraction techniques involve increased temperatures and are time-consuming, which could harm the molecules and their functions, having negative effects. The use of novel techniques has proved abundantly significant benefits, such us extraction time and temperature savings. These energy reductions (0.2 kW/h) and environmental benefits (200 g CO_2_/100 g extracted solid material) were noted for UAE, in comparison to maceration (6 kW/h and 3600 g CO_2_/100 g extracted solid material) [88]. Therefore, these techniques are consistent with extraction principles that are environmentally friendly [89]. Moreover, characteristics of the extracted compounds are also optimal. However, investigations have revealed some drawbacks related to the new extraction processes, due to safety (high noise/pressure levels), and the likely degradation of molecules under strong conditions (high ultrasound can depolymerize of hydrocolloids) [88]. To solve extraction limitations, a mix of extraction approaches has been suggested [89]. Most of these techniques can be scaled up to industrial scales or are currently being used successfully at industrial or semi-industrial scales. These techniques include advantages such as the ability to use water to produce larger yields of molecules. Nevertheless, despite the fact that the majority of novel technologies are frequently described as low-energy approaches, these findings should be confirmed using appropriate life cycle assessment methodologies, which assess these techniques’ efficacy for particular applications based on compound yields and energy and resource consumption [90]. Furthermore, depending on treatment conditions, unique extraction processes can cause a change in the conformation and structure of molecules, potentially altering them. Nevertheless, the adoption of emerging techniques, including their combined use, has shown promising results in terms of enhancing extraction yields and efficiency, and in the meantime, in reducing the processing time (Table 1). Future challenges include the need to scale up methods that are now being developed at the laboratory stage, so that they may be adapted to commercial needs.

## 4. Potential Use of Seaweed for Nutraceutical Applications

The most ancient information regarding the use of seaweed as food for therapeutical purposes goes back to ancient times in Japan. Even though many of the transcripts have been destroyed or lost, the properties of *Sargassum* sp. were investigated and compiled in Chinese medical literature “*Compendium of Materia Medica*”, written by Shizhen Li in 1578. While the *Compendium* also states that *Sargassum* sp. can soften hard lumps, dispel nodes, eliminate phlegm, induce urination in humans [91], treat fever, infections, laryngitis, and other diseases [92], the most ancient information on *Sargassum* focuses on its ability to treat thyroid-related diseases, such as goitre, and iodine deficiency disorders [93]. Vietnamese medicine frequently employs species from the *Eucheuma* and *Kappaphycus* (Rhodophyta) genera to reduce the incidence of tumors, ulcers, and headaches. 

Although recent researchers see *Sargassum* sp. as a suitable immunomodulator, due to its bioactive metabolites, which may improve immune function and inhibit thyroid growth caused by excessive iodine absorption, important information related to the treatment of thyroid-related conditions, such as goiter, as claimed for *Sargassum* sp. in traditional Chinese medicine, has not yet received enough research [94]. Asian cultures still consume seaweed for therapeutical applications, and this practice is widely spreading across the world as the beneficial properties of seaweed have been abundantly confirmed by scientists.

Currently, preclinical and clinical tests have demonstrated the efficacy of several biological activities of seaweed bioactive compounds. 

Meinita et al. [95] collected case studies conducted on seaweed and the treatment of chronic disease [96]. The research records a higher percentage of experiments conducted with brown seaweed (68%), followed by red seaweed (18%), and green seaweed (14%). The most extensively investigated species of brown seaweed were *Ecklonia*, *Sargassum*, and *Fucus* (they represent 21.3%, 20.2%, and 9%, of the total of the studies collected, respectively). The two species of red seaweed that have been examined the most for potential application in the treatment of chronic illnesses are *Gracilaria*, (20.8%) and *Gelidium* (16.7%). While, *Ulva* (47.4%), *Codium* (26.3%), and *Caulerpa* (47.4%, 26.3%, and 15.8%, respectively) the three species of green seaweed explored the most. Brown seaweeds have been the most researched in relation to cancer, diabetes, arthritis, neurodegenerative illnesses, obesity, osteoporosis, liver disease, and cardiovascular disease. Not many clinical trials have been performed, however, one was carried out to evaluate a seaweed extract formulation from *Fucus vesiculosis*, *Macrocystis pyrifera*, and *Laminaria japonica,* on osteoarthritis patients. According to the study, the formulation would ameliorate osteoarthritis symptoms in a dose-dependent way [97].

Manufacturers and consumers have shown a growing interest in incorporating functional components into daily diets in recent years. Moreover, the antioxidant and antimicrobial activities exhibited by seaweed compounds will ensure safety and delete the spoilage of food. The presence of flavonoids in the green algae *Ulva reticulata* and *Ulva* sp. (Chlorophyta) showed potential free-radical-scavenging ability [98,99]. Strong DPPH-radical scavenging activity has been measured in brown algae including *Eisenia bicyclis*, *Ecklonia cava*, and *Ecklonia kurome* [100], as well as for red algae *Callophyllis japonica* and *Gracilaria tenuistipitata* ethanolic extracts [101,102]. Seaweed polar lipids are now well recognized as essential phytochemicals that contribute and add usefulness and potential advantages for our health. Lopes et al. [103] set out to reveal the lipid profile of *Palmaria palmata* raised in an integrated multitrophic aquaculture (IMTA) to test its antioxidant properties. A total of 143 lipids were discovered, spanning numerous polar lipid groups such as phospholipids, glycolipids, and betaine lipids. It is important to note that eicosapentaenoic acid (EPA) accounts for more than half of the lipid content. One of the primary determinants of the antioxidant effectiveness of *Palmaria palmata* may be its level of EPA. Therefore, this finding suggests that this red macroalga could be used in the future as a source of EPA-rich lipids and antioxidant activity for functional foods [103].

The inclusion of seaweed molecules in food can contribute to ameliorate the lifestyle of people with certain medical conditions, as they possess interesting biological properties that guarantee health benefits. Seaweed, as a nutritional source, inhibits the growth of cancer cells, most likely due to its antioxidant characteristics. Antioxidants are clearly important in the later phases of cancer formation, as evidenced by the mechanisms of carcinogenesis promoted by oxidation activity. As a result, antioxidants are regarded as a viable method for regressing premalignant lesions and preventing cancer development [104]. Sulphated polysaccharide, derived from *Gracilaria lemaneiformis,* showed exceptional anti-cancer and immunomodulatory activity against transplanted H22 hepatoma cells in mice. Tumor growth was significantly slowed, splenocyte proliferation was boosted, macrophage phagocytosis was increased, and the number of IL-2 and CD8+ T cells in the blood increased [105]. The antiproliferative effect of κ-and λ-carrageenan extract from the red seaweed *Laurencia papillosa* has been demonstrated in vitro with human breast cancer cell line MCF-7 [106]. κ-carrageenan from *Kappaphycus alvarezii*, an edible seaweed, have been investigated for their antiproliferative activity. Results from the incubation of two LMWF of carrageenan with human colon cancer cells HCT116 revealed that these fractions may induce apoptosis via the ROS-mediated mitochondrial pathway by upregulating the latter, along with upregulating Bcl-2 and Bcl-xL, caspase3, and downregulating XIAP, an inhibitor of apoptosis. The investigated fractions could be incorporated in food to prevent colon carcinogenesis. Dietary behaviors influence the development of colorectal cancer; therefore, identifying dietary components that can help to prevent cancer could help people acquire healthier eating habits. LMWF soluble dietary fibers could be a potential additive in nutraceutical food, contributing to the efficacy of several health-promoting advantages as cancer treatment coadjutants. Exploring their health advantages would offer up new avenues for research in the nutraceutical field [107]. 

Antimicrobial and antioxidant properties of *Kappaphycus alvarezii* extracts were tested in both hot water and ethanolic extracts by Bhuyar et al. [108]. *Escherichia coli* and *Bacillus cereus* were used as pathogenic bacteria in the investigation of antibiotic activity. Both extracts’ antibacterial activity was more potent against *Bacillus cereus* than against *Escherichia coli*, suggesting that they might be able to maintain a healthy level of reactive oxygen species. Levoglucosenone, which has a highly functionalized chiral structure and can be used as a crucial intermediate in the development of biologically active compounds, and pyridinemethanol, a functional pyridine that is used as an intermediate in the pharmaceutical industry, were among the fatty acids found in both extracts; 1,2,5- Thiadiazole-3-carboxamide, which can be utilized as an antibacterial; and hexamethylcyclotrisiloxane, which is widely employed in medical, military aircraft, and other petrochemical sectors. Furthermore, GC-MS analysis revealed that considerable levels of levoglucosenone (48.9%) and 4-pyridinemethanol (28.21%) were found in hot water extract, suggesting that it may have antitumor potential [108].

A recent case study isolated k-carrageenan from the edible red alga *Hypnea musciformis* to assess antioxidant, antimicrobial, anticancer, and neuroprotective activities. Results revealed an antibacterial and antifungal action against *Staphylococcus aureus* and *Candida albicans*, respectively. The antiproliferative activity was tested on human breast cancer (MCF-7), human neuroblastoma (SH-SY5Y), and Balb/c 3T3 mouse fibroblast cell-lines. Carrageenan did not showed cytotoxic effect against MCF-7 and SH-SY5Y, while a reduction in proliferation has been identified. No significant effects have been detected for Balb/c 3T3 mouse fibroblast cells. *Hypnea musciformis* also showed neuroprotective action in SH-SY5Y cells, via modulating mitochondrial transmembrane potential and lowering Caspase 3 activity in 6-hydroxydopamine-induced neurotoxicity, but low antioxidant effect was detected [109]. According to this study, the edible red alga *Hypnea musciformis* has pharmacological potential, which may offer fresh perspectives on the creation of novel functional food, with promise for fighting microbes, cancer, and neuroprotection [109].

Among Asian seaweeds, the popular red edible seaweed *Gelidium amansii* is frequently utilized in Korea, Taiwan, China, and Japan, as a cuisine ingredient. Particularly appreciated in Taiwan and Japan, agar jelly is prepared from hot-water extracts of *Gelidum amansii* [110]. Galactose (23%) and glucose (20%) are particularly abundant in this red seaweed’s carbohydrate content [111]. Due to its easy and low-cost cultivation, *Gelidium amansii* is commonly involved in the manufacturing of agar [112]. Moreover, extracts of this alga showed that diabetic rats’ liver and plasma lipid levels could be lowered by supplementing high-cholesterol and high-fat diets [110]. Due to its numerous health benefits, *Gracilariopsis chorda* (Rhodophyta) is another popular seaweed in Korea, and it is also used as a food component [113]. In addition to Korea, France, Indonesia, Mexico, Morocco, Portugal, and Spain, also use *Gracilariopsis chorda* as a raw material to extract agar [114]. 

*Caulerpa lentillifera* (Chlorophyta) is cultivated and commercially sold in East Asian countries for use as both human and farm animal food [115]. Traditional uses for *Caulerpa lentillifera* extracts include the treatment of bacterial and fungal diseases, diabetes, rheumatism, and high blood pressure [116]. Recent research has shown that *Caulerpa lentillifera* extracts have the potential to become drugs or useful materials for the treatment of cancer and diabetes mellitus [117]. 

*Codium fragile* is a green edible seaweed that grows on the beaches of Asian regions and some coastal regions of Northern Europe. Traditional Korean medicine has utilized *Codium fragile* to treat enterobiasis, dropsy, and dysuria, and is currently used as a food ingredient. The potential of *Codium fragile* to create therapeutic meals has been suggested, as its compounds exhibit intriguing bioactive activities, including antioxidant, anticancer, anti-angiogenic, and anti-inflammatory activities [118]. 

Japanese seaweed-related food appears to be one of the first profitable industries in global trade because of its intriguing bioactive capabilities. However, consumption of seaweed is still not very widespread in other nations, likely because people are less aware of the health advantages of eating seaweed [119].

## 5. Conclusions and Future Perspective

In recent decades, the public’s attention to nutrition and health has grown due to the rising prevalence of chronic diseases, which are typically brought on by unreasonable lifestyle choices [120]. More individuals are focusing on improving their health through eating [121], which has led to the steady emergence of bioactive substances and functional food as hot issues [122]. The incorporation of seaweed in food introduces an innovative and functional food, which not only gives the right amount of nutrients and minerals to our organisms, but seaweed compounds possess interesting biological properties that can enhance and improve people’s health. 

To have sustainable production of nutraceutical food, industries must consider quality, cost, and eco-friendliness of the production processes. Seaweed-based products are based on seaweed’s bioactive compounds, which need to be extracted from seaweed. Before selecting the best extraction technique, several elements must be considered including price; time; quantity and kind of solvents; sustainability; and the potential for scaling up. The most typical extraction techniques for algal substances are traditional and less environmentally friendly. It is crucial to demonstrate the effectiveness and safety of new, sustainable extraction technologies through ongoing research, in order to decrease implementation cost, time, and pollution [42,55]. 

The present review suggests the consumption of seaweed-based food will ameliorate the quality of life for patients with health conditions. Therefore, further studies should be performed to evaluate the best extraction methods for target compounds; more assays need to be performed to evaluate the quality of compounds for the development of nutraceutical food.

## Figures and Tables

**Table 1:** Advantages and disadvantages of different extraction methods.

Extraction Method	Advantages	Disadvantages
Hot water/alkali extraction	Optimal rheological properties and purity of the extracted hydrocolloids	Involves high temperatures and very long extraction time
		Long extraction time and high temperature may affect extracted compounds’ functionalities
		Use of hazardous chemical solvents
		High cost of chemical solvents
Microwave-assisted extraction (MAE)	Use of water instead of chemical solvents	High temperature can deteriorate thermolabile compounds
	It provides locally heat raw materials, enhanced biomass digestion, reduced process time, solvent consumption, and costs	
	Extracted compounds possess good quality	
	It utilizes directly on fresh biomass from seaweed	
Ultrasound-assisted extraction (UAE)	Ability to achieve larger yield of extracts utilizing water	High noise levels involved (safety issues)
		Ultrasound might bring to depolymerization of compounds
	It increases extraction yield with lower extraction time	Due to the high cost of energy and equipment, UAE needs a large amount of capital to get started on an industrial scale
	Efficient, environmentally friendly, and low extraction processes. Low equipment expenses and maintenance, possibility to scale-up to industrial production, reduced number of process steps	UEA applications are still limited
	Extraction techniques used in food industry	
	Ability to obtain larger yield of extracts utilizing aqueous-based solvent	High-pressure involved (safety issue)
Pressurized solvent extraction (PSE)		High-pressure power can bring depolymerization of compounds
	It has high extraction performance, less solvent usage, quick extraction time, and does not imply the use of hazardous solvents	These processes might degrade labile compounds due to high temperature and pressure
		Scarcity of application on seaweed extractions
Enzyme-assisted extraction (EAE)	Ability to achieve larger yield of compounds utilizing water	Scarcity of application on seaweed extractions
	It is inexpensive, highly efficient, possibility to scale up, avoid the use of any harmful chemicals or organic solvents and it has shorter extraction time	
	It preserves the structural integrity of the target compounds extracted that exert important bioactivities suitable for cosmetic, nutraceutical and pharmaceutical industries	

## Data Availability

Not applicable.
